# A stemness-based signature with inspiring indications in discriminating the prognosis, immune response, and somatic mutation of endometrial cancer patients revealed by machine learning

**DOI:** 10.18632/aging.205979

**Published:** 2024-07-30

**Authors:** Xuecheng Pang, Yu Wang, Qiang Zhang, Sumin Qian

**Affiliations:** 1 Gynecology Department 2, Cangzhou Central Hospital, Cangzhou, Hebei, China; 2 Second Department of Anesthesia, Cangzhou Central Hospital, Cangzhou, Hebei, China

**Keywords:** endometrial cancer, random forest, stemness subtype, mRANsi, prognostic model

## Abstract

Endometrial cancer (EC) is a fatal gynecologic tumor. Bioinformatic tools are increasingly developed to screen out molecular targets related to EC. Our study aimed to identify stemness-related prognostic biomarkers for new therapeutic strategies in EC. In this study, we explored the prognostic value of cancer stem cells (CSCs), characterized by self-renewal and unlimited proliferation, and its correlation with immune infiltrates in EC. Transcriptome and somatic mutation profiles of EC were downloaded from TCGA database. Based on their stemness signature and DEGs, EC patients were divided into two subtypes via consensus clustering, and patients in Stemness Subtype I presented significantly better OS and DFS than Stemness Subtype II. Subtype I also displayed better clinicopathological features, and genomic variations demonstrated different somatic mutation from subtype II. Additionally, two stemness subtypes had distinct tumor immune microenvironment patterns. In the end, three machine learning algorithms were applied to construct a 7-gene stemness subtype risk model, which were further validated in an external independent EC cohort in our hospital. This novel stemness-based classification could provide a promising prognostic predictor for EC and may guide physicians in selecting potential responders for preferential use of immunotherapy. This novel stemness-dependent classification method has high value in predicting the prognosis, and also provides a reference for clinicians in selecting sensitive immunotherapy methods for EC patients.

## INTRODUCTION

Over the past decade, there has been a significant rise in the incidence of endometrial cancer (EC), making it one of the prominent gynecological malignancies among the three major types [1]. While 67% of patients are diagnosed with early-stage disease, resulting in an 81% 5-year overall survival (OS), the prognosis for those with stage IVA and IVB EC is considerably poorer, with 5-year OS rates of only 17% and 15%, respectively [2]. In 2020, there were a reported 417,367 newly diagnosed cases of EC worldwide, resulting in an estimated 97,370 deaths related to this cancer [3]. Prior research has shown that approximately 70% of endometrial cancer (EC) cases are diagnosed in postmenopausal women, while 15% of cases are found in premenopausal women [4]. Ultrasonography is a frequently employed method for the initial screening of EC, while magnetic resonance imaging (MRI) is considered the gold standard for preoperative pathological staging. The earliest sign of EC is abnormal vaginal bleeding [5]. The conventional treatment for EC involves a standard procedure, which includes hysterectomy and bilateral salpingo-oophorectomy, with or without lymphadenectomy. Subsequent adjuvant therapy is tailored to the patient’s risk factors. Nonetheless, in cases involving younger patients who have a strong desire to preserve their fertility, especially if they have not yet had children, a fertility-sparing approach is often necessary. This approach typically incorporates the use of oral or uterine local progestin in combination with GnRH-a or other regimens, along with regular hysteroscopic biopsies [6]. Research findings indicate that inhibiting PROM2 expression results in heightened sensitivity to paclitaxel, leading to decreased IC50 values and reduced proliferation in endometrial cancer. Moreover, the knockdown of PROM2 has been observed to promote apoptosis in endometrial cancer cell lines [7]. There is a certain relationship between gene expression and resistance to chemotherapy drugs. For example, in head and neck tumors, multiple genes are associated with chemotherapy drug resistance [8, 9]. Recently, bioinformatics has played an increasingly essential role in predicting survival. Bioinformatics analysis and computational approach had high accuracy in predicting the prognosis of cancer patients [10]. While a majority of EC cases are identified in the early stages, leading to a relatively positive prognosis thanks to early detection, there remains a subset of approximately 28% of patients who succumb to the disease. Their deaths are typically attributed to distant metastasis and recurring instances, which frequently result in a limited response to conventional therapies [11]. However, patients with the same degree of progression can show different prognoses and treatment responses [12]. Hence, it becomes paramount to underscore the molecular alterations in order to forecast the occurrence of metastasis and relapse in EC, while also ensuring the vigilant monitoring of EC patients’ prognoses. In the context of solid malignant tumors, the pivotal role played by cancer stem cells (CSCs) cannot be overstated, as they significantly contribute to disease progression, recurrence, and the development of drug resistance [13]. Moreover, CSCs facilitated immunosuppression, immune evasion, tumor metastasis, and resistance to treatments through their interactions with immune cells [14]. In order to delve deeper into the unique characteristics of cancer stem cells, Malta and colleagues harnessed cutting-edge deep learning methods. They crafted a scoring system using the One-Class Logistic Regression (OCLR) machine learning algorithm to gauge the resemblance between tumor cells and diverse stem cell types sourced from the Progenitor Cell Biology Consortium (https://www.synapse.org/pcbc). This endeavor led to the development of two distinct stemness indices: the DNA expression-based stemness index (mDNAsi) and the mRNA expression-based stemness index (mRNAsi). The perpetuation of tumor growth hinges on an exceedingly limited population of self-renewing stem cells. Research findings have uncovered a robust association between mRNAsi and the prognosis of EC, offering novel insights into the prediction of EC outcomes [15].

As our comprehension of the biology of EC has advanced, it is now evident that various histologic types of EC should not be regarded as a singular disease. Alternative treatments aimed at specific biological subsets of EC have made substantial progress [16]. Despite the availability of multiple markers for the isolation and characterization of cancer stem cells (CSCs) in endometrial cancer (EC), such as cluster of differentiation (CD)44, CD117, aldehyde dehydrogenase (ALDH), CD133, and CD24 [17], the comprehensive assessment of a tumor’s overall stemness still poses a significant challenge [18]. In the ever-evolving field of cancer treatment, the role of the tumor microenvironment (TME) and immune checkpoints (ICs) holds paramount importance in the realm of oncology research. The introduction of immune checkpoint inhibitors, such as programmed death-1 receptor (PD-1) and cytotoxic T-lymphocyte antigen 4 (CTLA-4) inhibitors, has propelled immunotherapy into a promising frontier for managing cancer. This therapeutic approach has exhibited remarkable clinical effectiveness in a wide range of solid tumor types [19]. Additionally, molecular alterations such as POLE-mutated or microsatellite instability (MSI) are associated with a big number of tumor-infiltrating immune cells (TICs), which might be appropriate candidates for PD-1/PD-L1 immune therapies [20]. While research has highlighted the crucial role of immunotherapy in EC, the exact molecular mechanisms that underlie its effectiveness remain enigmatic. Therefore, it is essential to further explore the immune-mediated molecular intricacies that are unique to EC, with the goal of unveiling more potent and effective therapeutic strategies.

In this study, differential analyses were conducted in patients with EC to evaluate their stemness index. Consequently, based on distinct mRNAsi features, EC patients were classified into two subgroups with distinct survival outcomes, somatic mutations, and clinicopathological characteristics. Subsequently, a comprehensive analysis was employed to examine the distinctions within the tumor microenvironment, genomic variations, and patterns of immune response among patients with EC subtypes I and II. By integrating multiple machining learning, hub genes were selected and prognostic risk signature was developed and verified by patients in Cangzhou Central Hospital. Our research aims to establish a new molecular classification based on stem cells to help doctors predict the individual survival of EC patients and make better treatment choices.

## MATERIALS AND METHODS

### Data acquisition and clinical information

The information utilized encompasses the fragments per kilobase of FPKM (transcript per million mapped reads) standardized sequencing dataset, as well as the associated clinical data (including age, stage, histological type, menopausal status, grade, cancer status, lymph node metastasis, survival information, and other clinical particulars Supplementary File 1) for both EC samples and normal samples, was sourced from The Cancer Genome Atlas (TCGA) website (https://portal.gdc.cancer.gov/).

### Sample collection of Cangzhou Central Hospital cohort

The nomogram’s predictive accuracy was confirmed in the testing cohort, which comprised 24 surgically patients at the Obstetrics and Gynecology Department, Cangzhou Central Hospital. For this cohort, both RNA sequencing results and clinical data were accessible. Samples for this validation were obtained from patients treated between January 2008 and December 2012. The protocols for total RNA isolation and reverse transcription-quantitative PCR were consistently applied in line with established procedures [21]. This study was approved by the Ethics Committee of Cangzhou Central Hospital.

### Differential analysis of the high and low mRNAsi groups

mRNAsi were obtained from previous research [22], which was based on a OCLR machine learning algorithm. We acquired the mRNAsi of EC patients and integrated it with TCGA data on EC. This integration was achieved using a Perl merge script, with unmatched cases being removed. The stemness indexes served as indicators of the likeness between tumor cells and stem cells, with mRNAsi specifically capturing transcriptomic stemness characteristics.

### Identification of the stemness-based molecular classification of EC patients

We conducted survival analysis to evaluate the prognostic implications of distinct stemness subtype groups. Additionally, both univariate and multivariate Cox regression analyses were conducted to assess whether the prognostic significance of the stemness subtype remained statistically significant when considering other clinicopathological variables. These analyses were carried out using the “ConsensuClusterPlus” package, and we repeated these steps 1000 times to ensure the robustness and stability of the classification [23]. Unsupervised clustering analysis was utilized to identify DEGs and to classify patients into distinct clusters for further investigation. The ideal number of clusters and their robustness were assessed using the consensus clustering algorithm Supplementary File 2 [24].

### Immune cell infiltration and the tumor microenvironment analysis

CIBERSORT is a deconvolution method for expression matrices of immune cell subsets [25]. Moreover, immune scores of different subgroups were calculated with the package “estimate”, and plot histograms of differences in immune scores, stromal scores, ESTIMATE scores, and tumor purity of each EC tumor sample Supplementary File 3 [26]. Tumor Mutational Burden (TMB) for each tumor sample was quantified as the number of mutated bases per million bases, encompassing missense mutations, nonsense mutations, frameshift mutations, and other types of mutations. We calculated TMB values for each sample by Perl scripts, considering the number of variants across the human exome’s length (38 million bases).

### Construction and validation of the stemness subtype predictor by multiple machine learning methods

The 514 EC patients were randomly divided into training (*N* = 343) and testing (*N* = 171) sets in a 2:1 ratio. Initially, within the training set, we utilized the least absolute shrinkage and selection operator (LASSO) regression, Random Forest (RFB), and Cox regression analyses to identify the most significant group-related features by computing importance scores for each variable using the “glmnet,” “randomForest,” and “cox” packages in R [27, 28]. We employed the “survival” package in R to conduct univariate Cox proportional hazard regression analysis, aiming to identify stemness-related genes significantly associated with the overall survival (OS) of EC patients within the TCGA cohort. The most crucial stemness subtype-related genes, identified through the intersection of results from LASSO Supplementary File 4, Random Forest (RF), and Cox regression analyses, were visually represented using a Venn diagram. Finally, we carried out multivariate cox regression analysis on these critical genes to construct the predictive model, referred to as the ‘Stemness Subtype Predictor’ [27]. “We utilized ROC curve analysis to establish the optimal cutoff values for distinguishing between different subtypes and evaluated performance metrics such as the AUC, sensitivity, specificity, and accuracy. Following this, we assessed the predictive capabilities of the stemness subtype predictor in a test set derived from our hospital cohort, employing a methodology akin to that employed in the previously studied cohorts.”

### Statistical analysis

In the presentation of descriptive statistics, we employed ‘mean ± standard deviation’ for continuous variables following a normal distribution, and ‘median (range)’ for continuous variables exhibiting abnormal distribution. Categorical variables were represented by counts and percentages. For all statistical analyses, we employed R statistics software (version 3.6.1 Supplementary File 5). We evaluated the relationship between mRNAsi and diverse clinicopathological characteristics using the Chi-square test. The correlation between mRNAsi and tumor-infiltrating immune cells was determined using Spearman’s correlation. To assess the prognosis of endometrial cancer, we conducted Cox regression analysis. Kaplan-Meier method was used to analyses the difference in OS between different subtypes. *P* < 0.05 was considered statistically significant.

### Data availability statement

The data underlying this article are available in the article and in its online supplementary material.

## RESULTS

### Distribution of mRNAsi in patients with different clinicopathological features and mutation status

**Figure 1:**
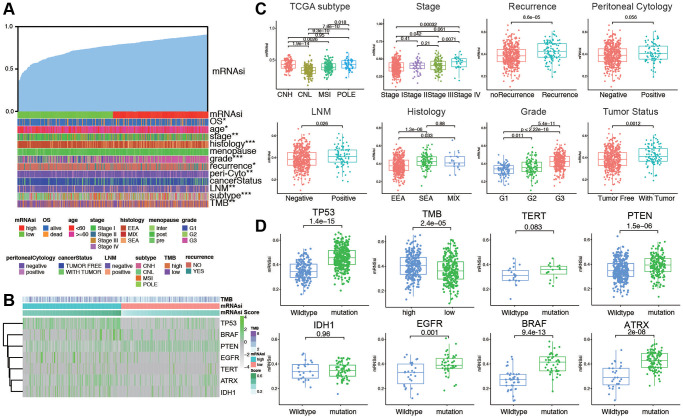
**The clinical and molecular features associated with the stemness index (mRNAsi) in EC patients.** (**A**) An overview of the association between mRNAsi and clinicopathological features of patients. Columns represented samples ranked by mRNAsi from low to high (top row), and rows represent known clinical and molecular characteristics associated with mRNAsi. (**B**) Heatmap of the association between TMB and somatic mutation status of the most popular biomarkers of EC. (**C**) Distribution of mRNAsi in different clinicopathological features including TCGA subtypes, stage, recurrence, peritoneal cytology, LNM, histology, grade, and tumor status displayed by boxplots. (**D**) Distribution of mRNAsi in different somatic mutation status of the biomarkers including TP53, TMB, TERT, PTEN, IDH1, EGFR, BRAF, ATRX.

The clinicopathological characteristics of the patients are shown in Supplementary Table 1. We arrange the patients from low to high according to their mRNAsi values. The corresponding clinicopathological changes are shown in Figure 1A by heatmap. It can be seen that OS and other factors show significant differences with the increase of mRNAsi value. In addition, we also explored the mutation of TMB and several genes in the high and low mRNAsi groups. The results showed that there were significant differences in mutations of TMB among different mRNAsi groups (Figure 1B). Next, we explored the distribution of mRNAsi in different clinicopathological features and gene mutation subgroups. The results showed that mRNAsi had significant differences in most clinicopathological features (TCGA subtype, stage, recurrence, lymph node metastasis, histology, grade, tumor status, age, living status, and menopausal status, Figure 1C and Supplementary Figure 1). The mRNAsi score was higher in worse pathological features. On the other hand, mRNAsi has a higher score in gene mutation groups, including TP53, PTEN, EGFR, BRAF, ATRX, and high TMB group. Above all, these results indicate that higher stemness index is reflected with worse prognosis and mutation status (Figure 1D).

### Construction of EC grouping based on ssGSEA and immune microenvironment landscape of stemness index

**Figure 2:**
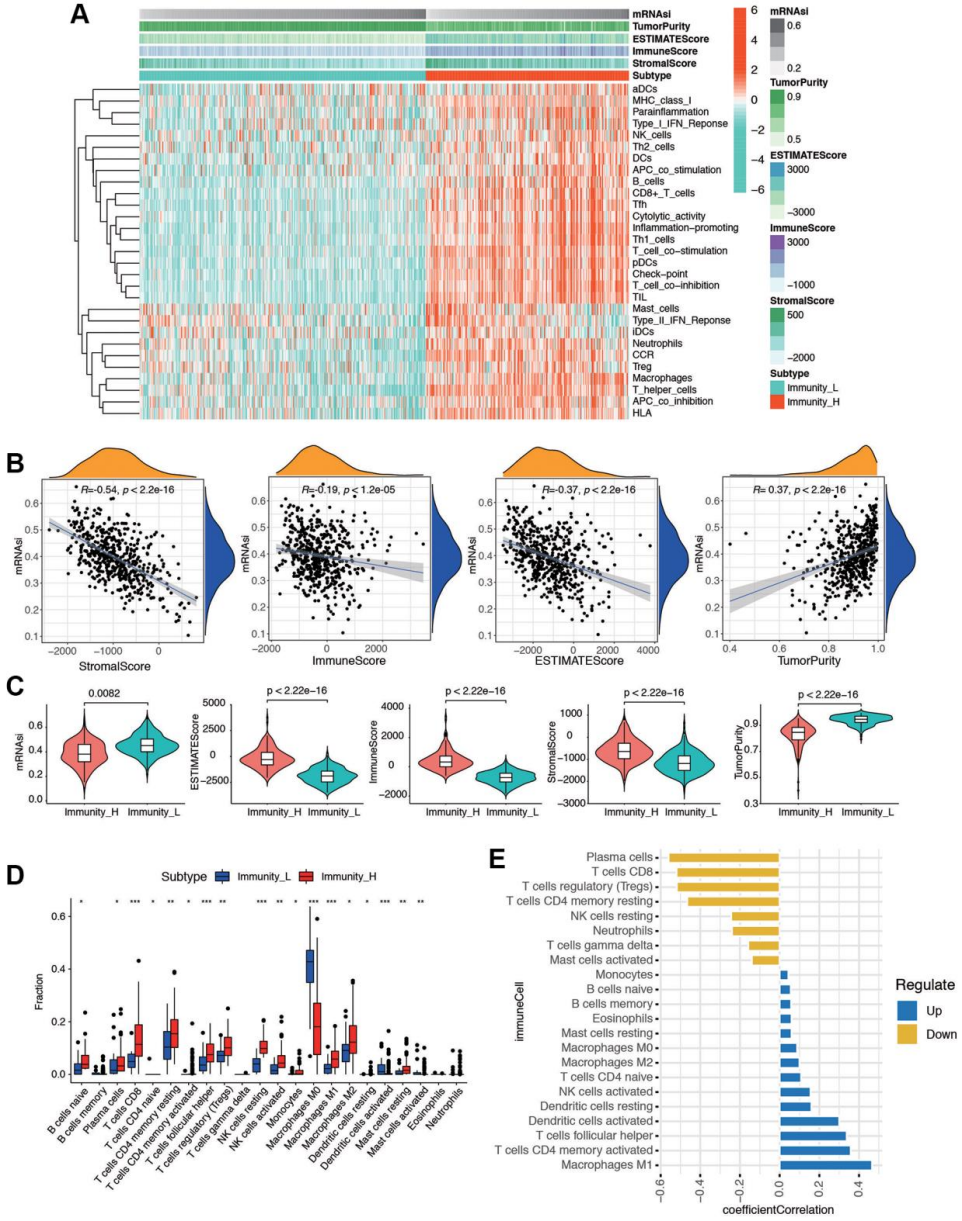
**The tumor immune microenvironment patterns and immunogenomic features of EC associated with the mRNAsi.** (**A**) The immune cells were highly expressed in the cluster 1, which was named as the high immune cell infiltration group (Immunity_H), and the low expression in the cluster 2 group was named as the low immune cell infiltration group (Immunity_L). Using ESTIMATE's algorithm, the tumor purity, ESTIMATE score, immune score, and stromal score of each sample gene was displayed together with the grouping information. (**B**) Correlation analysis between mRNAsi and different kinds of score, including stromal score, immune score, ESTIMATE score, and tumor purity. (**C**) The violin plot showed that there was a statistical difference in Tumor Purity, ESTIMATE Score, Immune Score and Stromal Score between the two groups (*p* < 0.01). (**D**) Different distributions of tumor-infiltrating cells in two immunity clusters (^***^p < 0.001, ^**^p < 0.01, ^*^p < 0.05). (**E**) Correlation analysis between immune cells and mRNAsi. Blue bars meant correlation coefficient >0, and yellow bars meant correlation coefficient <0.

We evaluated the immune status of tumor samples by employing the ssGSEA method on the transcriptomes of TCGA endometrial cancer specimens. This assessment incorporated 29 immune-related pathways and assessed the presence of infiltrating immune cells to estimate the immune profile of EC tissues (Figure 2A). The total TCGA cohort were clustered into 2 subgroups (low immunity: 221 samples, and high immunity: 293 samples) by applying unsupervised consensus clustering analysis (Supplementary Figure 2A). There was significant distinction existed on the transcriptional profile among these two immunity modification clusters (Supplementary Figure 2B). By stratifying the TCGA dataset into low and high immunity groups through unsupervised consensus clustering analysis, we categorized patients into two distinct subgroups, namely ‘immunity_H’ and ‘immunity_L.’ It was evident that the ‘immunity_H’ signature was linked to a more favorable prognosis, while the ‘immunity_L’ group exhibited worse survival outcomes (Supplementary Figure 2C). Several parameters were applied to estimate the immune infiltration profiles, including tumor purity, ESTIMATE score, immune score, and stromal score. The distribution of these scores is obviously different in immune subgroups (Supplementary Figure 2D). In addition, the low immunity group exhibited significantly lower HLA related gene set expressions (Supplementary Figure 2E). The distinction between the two immune subtypes may be attributed to the intricate nature of the tumor microenvironment (TME). To investigate the biological disparities between these clusters, we employed the ssGSEA algorithm to assess the prevalence of 28 distinct immune cell types within the immune-infiltrated microenvironment of EC. The findings indicated a significantly higher level of immune infiltration in ‘Immunity_H’ compared to ‘Immunity_L’ cluster, as evidenced by a more pronounced activation of immune response-related cells. Next, we investigated the association between mRNAsi and ESTIMATE-related scores. As shown in Figure 2B, mRNAsi was evaluated and found to be significantly negatively correlated with stromal score (*p* < 2.2e-16), immune score (*p* < 1.2e-05), and ESTIMATE score (*p* < 2.2e-16) in EC. Moreover, the results also revealed that the mRNAsi had an evident positive correlation with tumor purity (*p* < 2.2e-16). Then the mutation score and ESTIMATE score were compared between immunity_H and immunity_L groups. According to the results, the immunity_H had an obviously lower mRNAsi, tumor purity score, and higher ESTIMATE score, immune score, and stromal score compared with the immunity_L group in the dataset (*p* < 2.2e-16, Figure 2C). To explore if the high or low immunity status could affect the status of tumor immune microenvironment, tumor-infiltrating immune cells (TIICs) between different immunity groups were compared. The results exhibited that no significant differences were observed between two groups in abundance of the rest immune cells (Figure 2D). As shown in Figure 2E, the stemness index was significantly positively correlated with Macrophages M1 (R = 0.46, *p* < 0.01), activated CD4 T memory cells (R = 0.35, *p* < 0.01), T cells follicular helper (R = 0.34, *p <* 0.01), and activated dendritic cells (R = 0.29, *p* < 0.01); meanwhile the stemness index was significantly negatively correlated with plasma cells (R = −0.56, *p* < 0.01), CD8 T cells (R = −0.52, *p* < 0.01), Tregs (R = −0.51, *p* < 0.01), and resting CD4 T memory cells (R = −0.47, *p* < 0.01). These results indicated that immunity subgroups could have strong correlation with TME in EC.

### Differentially expressed genes analysis of mRNAsi and corresponding mutational features

**Figure 3:**
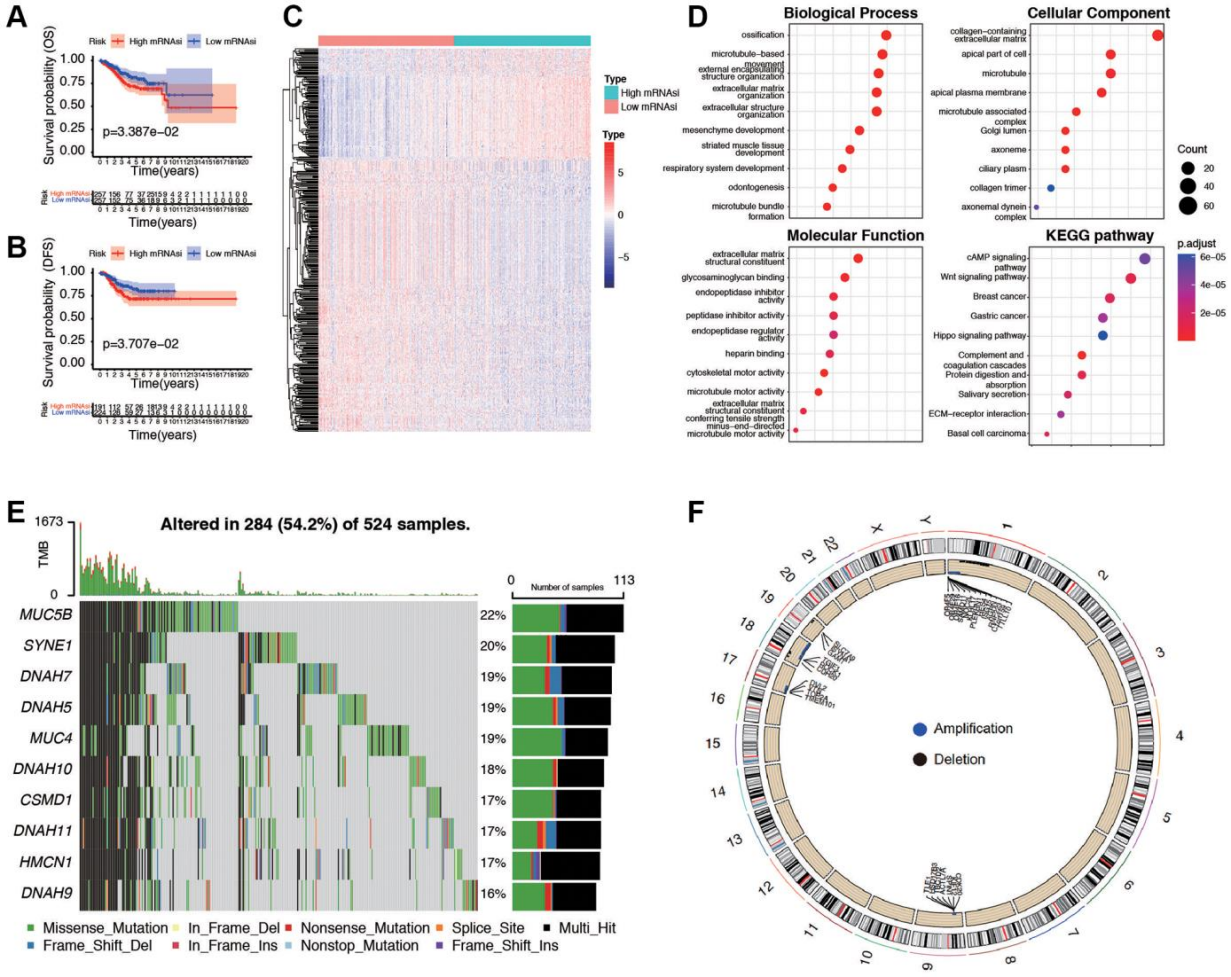
**Evaluation and differential expression analysis between low and high mRNAsi groups.** (**A**, **B**) Overall Survival curve and Disease-Free Survival curve of patients in low and high mRNAsi group. (**C**) Heatmap of differentially expressed genes (DEGs) between the mentioned two groups. (**D**) GO and KEGG functional analysis of DEGs. (**E**) Landscape of the top ten mutation DEGs profiles in EC samples. Mutation information of each gene in each sample was shown in the waterfall plot, where different colors with specific annotations at the bottom meant the various mutation types. The bar plot above the legend exhibited the number of tumor mutation burden (TMB). (**F**) The differential analysis of copy number variations between low and high mRNAsi groups was visualized by Circos plot, which revealed that compared with the low mRNAsi group. Blue dots represented amplifications, black dots represented deletions, and grey dots represented no significant CNAs.

Initially, a Kaplan-Meier curve was generated to examine the impact of mRNAsi values on the prognosis of EC patients. It was observed that patients with lower mRNAsi values experienced an extended overall survival (*p* = 0.033) and DFS (*p* = 0.037, Figure 3A, 3B), progression-free survival (PFS, *p* = 0.081, and disease-specific survival (DSS, *p* = 0.049, Supplementary Figure 3A, 3B). To further find out novel genes that play essential roles in EC microenvironment associated with stemness index, we grouped the samples based on the median of the mRNAsi, and then we conducted a differential analysis between the high and low-mRNAsi samples. The DEGs were displayed in the heatmap (Figure 3C) and volcano (Supplementary Figure 3C). The results showed that there were 1,290 DEGs between high and low mRNAsi groups, including 117 significantly upregulated genes and 1,173 significantly downregulated genes. GO and KEGG pathway analysis of DEGs exhibits intriguing results. In GO functional analysis, microtubule-based movement, extracellular matrix structural constituent, and collagen-containing extracellular matrix were enriched. The expression levels of the correlated genes in the enriched KEGG pathways are also displayed, including cAMP signaling pathway, Wnt signaling pathway, and Hippo signaling pathway (Figure 3D). Moreover, the mutation characteristics of DEGs in each EC sample are visually represented in a waterfall plot (Figure 3E), allowing for the analysis of distinct mutation types associated with individual genes contributing to EC progression. The rest of the mutation analysis are shown in Supplementary Figure 3D–3I. Comparing the two clusters, the differential analysis of copy number variations showed that, in the high mRNAsi group, 20 (16.7%) genes had significant amplifications, and 11 (7.2%) genes had significant deletions, in contrast to the low mRNAsi group (Figure 3F). All these results revealed that different mRNAsi subgroups had diverse survival and DEGs from high or low mRNAsi group might play a pivotal role in progression and somatic mutation in EC patients.

### Identification of tumor subtypes based on DEGs from mRNAsi using consensus clustering

**Figure 4:**
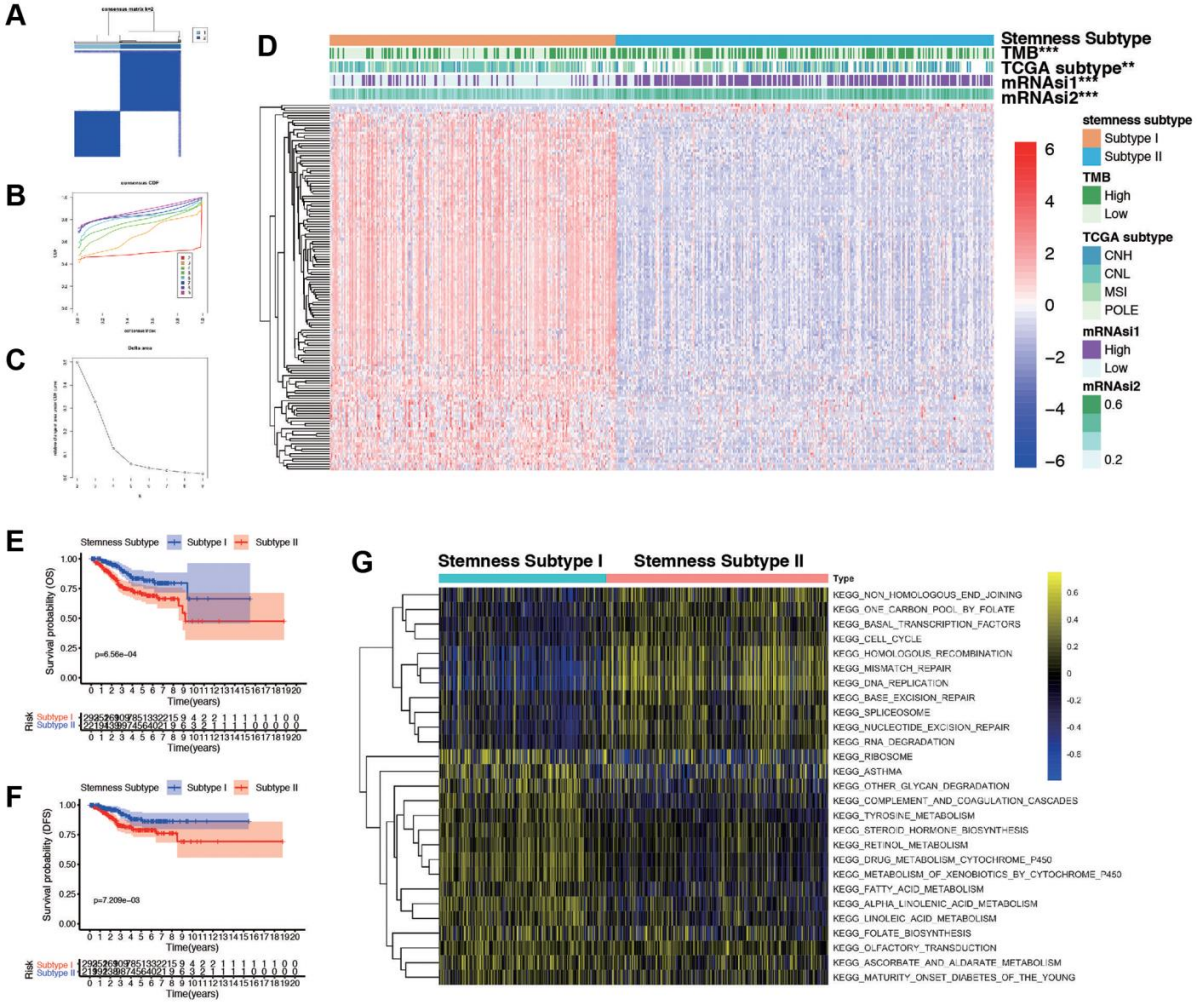
**Consensus clustering based on the DEGs and assessment of the Stemness Subtypes.** (**A**) Consensus clustering matrix for EC patients for DEGs in EC. (**B**) Consensus clustering distribution function (CDF). (**C**) Relative changes in the area under the CDF curve. (**D**) The heatmap shows the of 145 DEGs (including 3 up-regulated and 142 down-regulated genes) between different Stemness Subtypes and the clinical characteristics (TMB, TCGA subtypes, continuous variable of mRNAsi and categorical variable) in the TCGA database. (**E**, **F**) Survival curve of patients in different subtypes. Patients in subtype I had a promising prognosis in both OS and DFS. (**G**) Thermogram shows the activation state of KEGG pathways in different Stemness Subtype I and II after processing by GSVA. The yellow node represents high enrichment scores, and the blue node represents low enrichment scores, *p* < 0.05.

ConcensusCluster analysis was utilized to classify the EC samples based upon the DEGs from high and low-mRNAsi subgroups. As depicted in Figure 4A–4C and, we determined that selecting k = 2 was a sound decision, given the increase in cluster stability from k = 2 to 10. Additionally, we employed PCA analysis to explore the features of DEGs, using the k = 2 classification among EC patients (Supplementary Figure 4A). Heatmap was plotted to show the distribution of gene expression and mutation information, including TMB, TCGA subtypes, and mRNAsi are significantly different in two subtypes (Figure 4D). High TMB, high mRNAsi, and worse TCGA subtypes are enriched in subtypes II. Furthermore, we found that the two subtypes were associated with OS (*p* = 6.56e-04, Figure 4E), DFS (*p* = 7.21e-03, Figure 4F), and PFS (*p* = 1.66e-02, Supplementary Figure 4B), but not associated with DSS (*p* = 3.11e-01, Supplementary Figure 4C). To further explore the biological behaviors between distinct subtypes, we conducted GSVA and found that stemness subtype II presented enrichment pathways associated with tyrosine metabolism, steroid hormone biosynthesis, retinol metabolism, and fatty acid metabolism. Stemness Subtype I tumors mainly correlated with basal transduction factors, cell cycle, mismatch repair, and RNA degeneration (Figure 4G). According to these implications, the mRNAsi-related DEGs could categorized patients into two subtypes, and patients in two subtypes had diverse characteristics. Differentiating these functions of patients may occur in GSVA related results.

### CNA burden, TMB, and clinical features in different stemness subtypes

**Figure 5:**
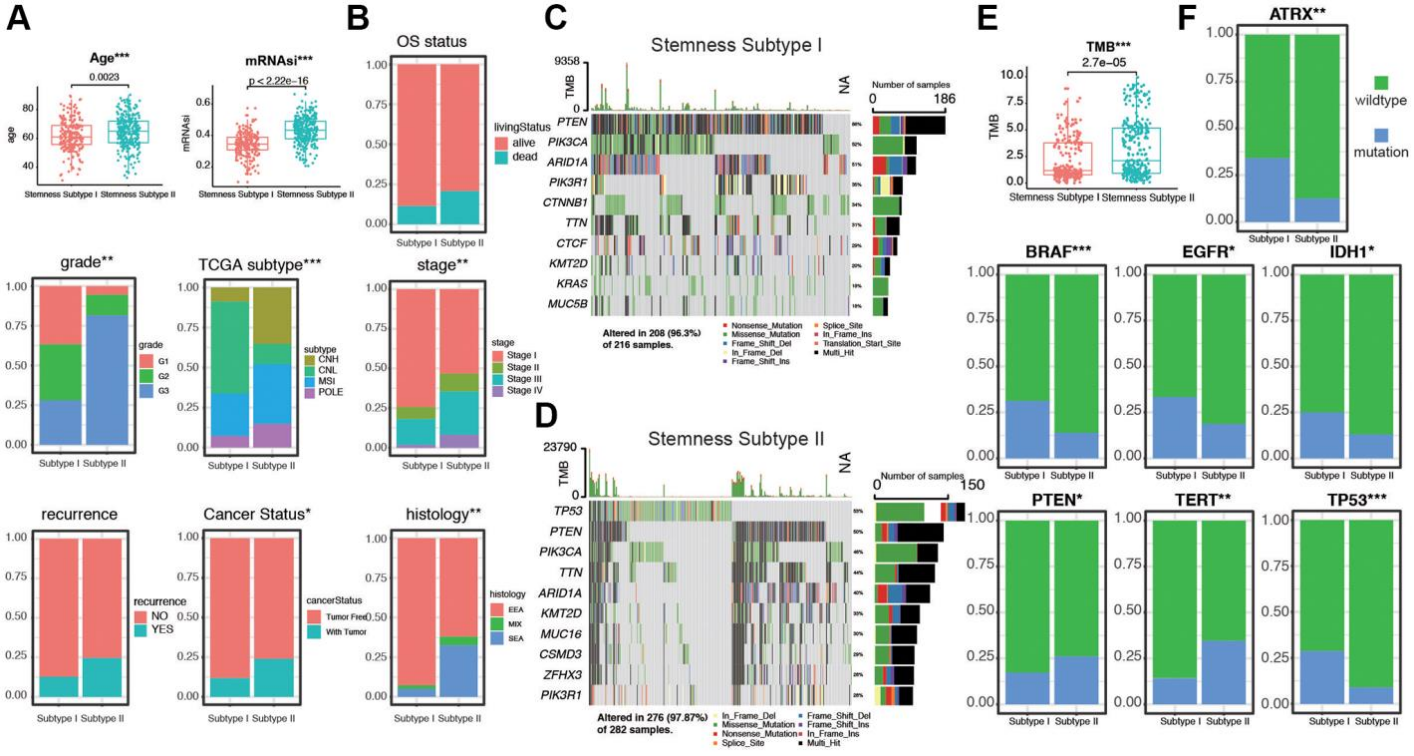
**Validation of the Stemness Subtype classification and exploration of the relevant clinical features and somatic mutational characteristics.** (**A**, **B**) Differences in clinical features between distinct Stemness Subtypes in TCGA cohorts. (**C**, **D**) The waterfall plot of tumor somatic mutation established by those with Stemness Subtype I and Stemness Subtype II. Each column represented individual patients. The upper bar plot showed TMB, the number on the right indicated the mutation frequency in each gene. The right bar plot showed the proportion of each variant type. (**E**) Distribution of TMB between two subtypes. (**F**) The comparisons of mutational status of ATRX, BRAF, EGFR, IDH1, PTEN, TERT, and TP53 promoter between Stemness Subtype I and II. (^*^p < 0.05; ^**^p < 0.01; ^***^p < 0.001; Kruskal-Wallis value).

To further explore the relationship between stemness subtypes and clinicopathological characteristics, we compare different clinical features in Stemness Subtype I and II. As shown in Figure 5A, patients in the Stemness Subtype I group were significantly lower in age (62.5 ± 22.4 versus 64.9 ± 24.1 years, *p* = 0.0023) and mRNAsi (0.35 ± 0.22 versus 0.44 ± 0.25, *p* < 2.22e-16) than those in the Stemness Subtype II group. The clinical factors in Stemness Subtype I was significantly different from that in the Stemness Subtype II group, such as grade, TCGA subtypes, stage, cancer status, and histological types (Figure 5B). However, the distribution of stemness subtypes were not correlated with OS status, recurrence, LNM, and peritoneal cytology (Supplementary Figure 5A, 5B). As shown in Figure 5C, 5D, subtype I group presented less extensive tumor mutation burden than the subtype II group. The box diagram of each color represents a kind of mutation (Supplementary Figure 5C, 5D). The value of TMB is much higher in Stemness Subtype groups (*p* = 2.7e-05, Figure 5E). We then compare the mutation occupation of key genes for EC. The same situations were observed for ATRX, BRAF, EGFR, IDH1, and TP53, indicating that the mutation frequencies of ATRX (I versus II, 30.8% versus 12.5%; *P* < 0.01), BRAF (I versus II, 29.1% versus 13.2%; *P* < 0.001), EGFR (I versus II, 30.2% versus 17.4%; *P* < 0.05), IDH1 (I versus II, 25.1% versus 13.2%; *P* < 0.05), and TP53 (I versus II, 27.5% versus 9.2%; *P* < 0.001) in the Stemness Subtype II group were significantly higher. Whereas, mutation frequencies of PTEN (I versus II, 16.3% versus 26.8%; *P* < 0.05) and TERT (I versus II, 13.3% versus 34.8%; *P* < 0.01) between the two subtypes in the Stemness Subtype I group were significantly higher (Figure 5F). Finally, we proved that the stemness subtype groups are diverse in clinicopathological features and key somatic mutations, which may indicate us these mutational genes play an essential in the progression derived from stem cells in EC.

### Distinct immunogenomic patterns and functions in two stemness subtypes

**Figure 6:**
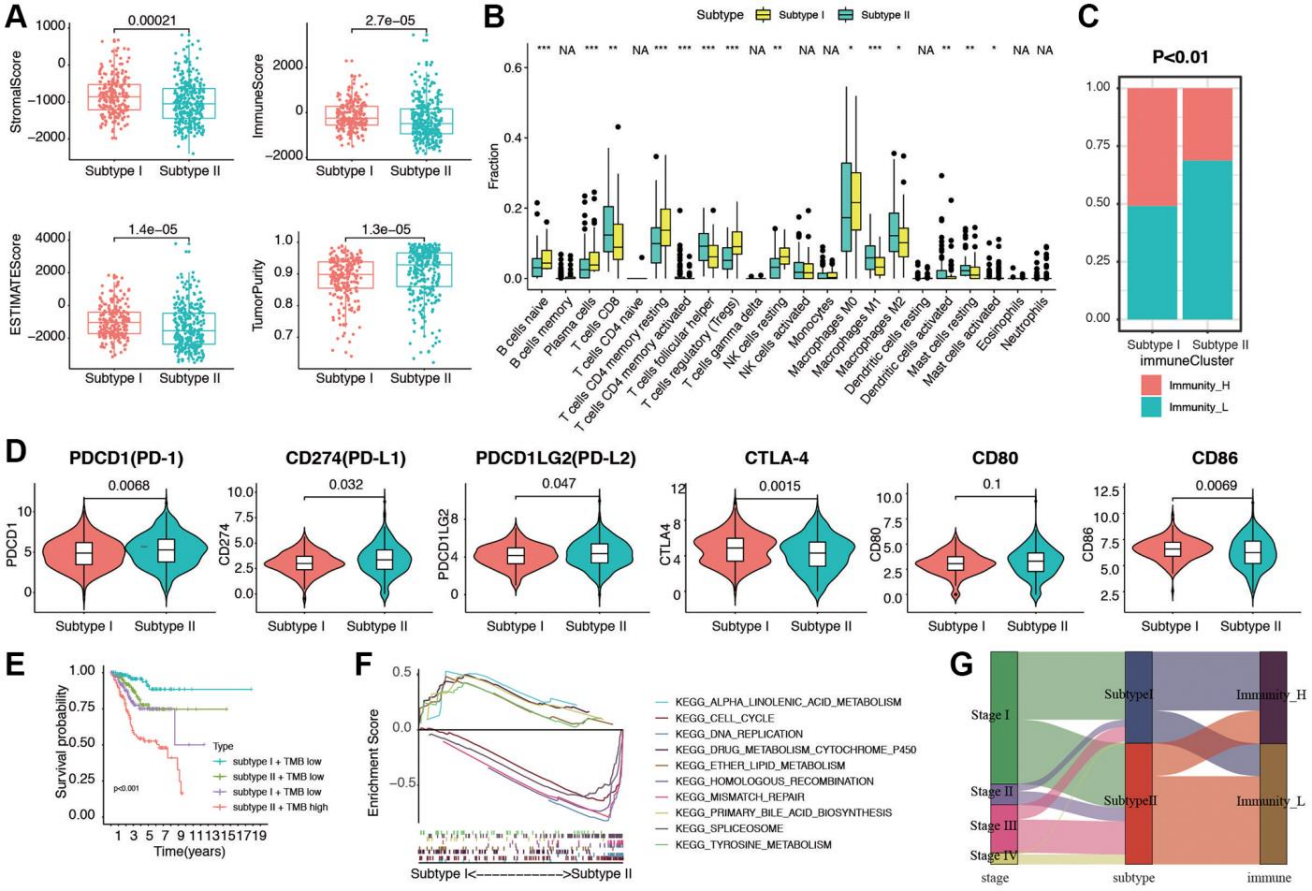
**Evaluation of immunogenomic patterns and functional analysis in two stemness subtypes.** (**A**) Different stromal score, immune scores, ESTIMATE scores, and tumor purity in two subtypes. (**B**) Different distributions of 22 kinds of tumor-infiltrating cells in two subtypes (^*^p < 0.05; ^**^p < 0.01; ^***^p < 0.001). (**C**) Different proportions of high and low immunity tumors in two stemness subtype. (**D**) The expression levels of PD-1, PD-L1, PD-L2, CTLA-4, CD80 and CD86 in Stemness Subtype I and II. (**E**) Survival analyses for subgroup patients stratified by both stemness subtype and TMB using Kaplan-Meier curves. (*p* < 0.001, Log-rank test). (**F**) GSEA showed the significantly enriched KEGG pathways based Stemness Subtype I and II. (**G**) Fractions of EC patients were shown in the form of a Sankey map according to different classifications (clinical stage: I/II/III/IV; stemness subtypes: I and II; immunity: high and low).

Next, ESTIMATE score and purity score in two stemness subtypes were compared. As shown in Figure 6A, stromal score, immune score, and ESTIMATE score were much higher in subtype I group (all *p* < 0.05). In contrast, tumor purity score was much higher in Subtype II group (*p* = 1.3e-05). To investigate the correlation between TIICs and stemness subtypes in EC, we first used CIBERSORT to calculate infiltration of 22 immune cells in the EC cases. Then, we compared the infiltration of 22 immune cells in stemness subtype I and II groups. The difference analytical results showed that naive B cells, plasma cells, CD8 T cells, T cells CD4 memory resting, T cells CD4 memory activated, T cells follicular help, Tregs, NK cells resting, Macrophages M0/M1/M2, Mast cells resting, Mast cells activated were significantly different in two groups (Figure 6B). Furthermore, consistent with the findings of previous studies, the proportion of immunity_H decreased from Stemness Subtype I (49.3%) to Stemness Subtype II (31.3%, Figure 6C). The part of high TMB group occupied more in Stemness Subtype II group (Supplementary Figure 6). In addition, to investigate the immunotherapy and response in two stemness subtypes, we compare the expression of related genes in two groups (Figure 6D). We then combined the subtype with TMB score, and divided the cohort into for subgroups according to subtypes and low/high TMB score. The results showed that patients in subtype I and low TMB subgroup had the best prognosis, and patients from subtype II, high TMB subgroup tended to survive shorter than the patients from other subgroups (Figure 6E). Next, GSEA was then performed, subtype I was enriched in cell cycle, DNA replication, and ether lipid metabolism, and subtype II patients were enriched in mismatch repair, primary bile acid biosynthesis, and tyrosine metabolism (Figure 6F). We visualized a Sankey map to illustrate the connections among stage, subtype, and immune status. The results indicated that patients in stage IV were primarily associated with Stemness Subtype II and displayed lower immune infiltration (Figure 6G). These intriguing findings underscore that Stemness Subtype I tumors exhibit comparatively low levels of immune infiltration and high tumor purity, while also having relatively elevated immune activity.

### Construction and validation of predictive prognostic model integrating multiple machine learning algorithms

**Figure 7:**
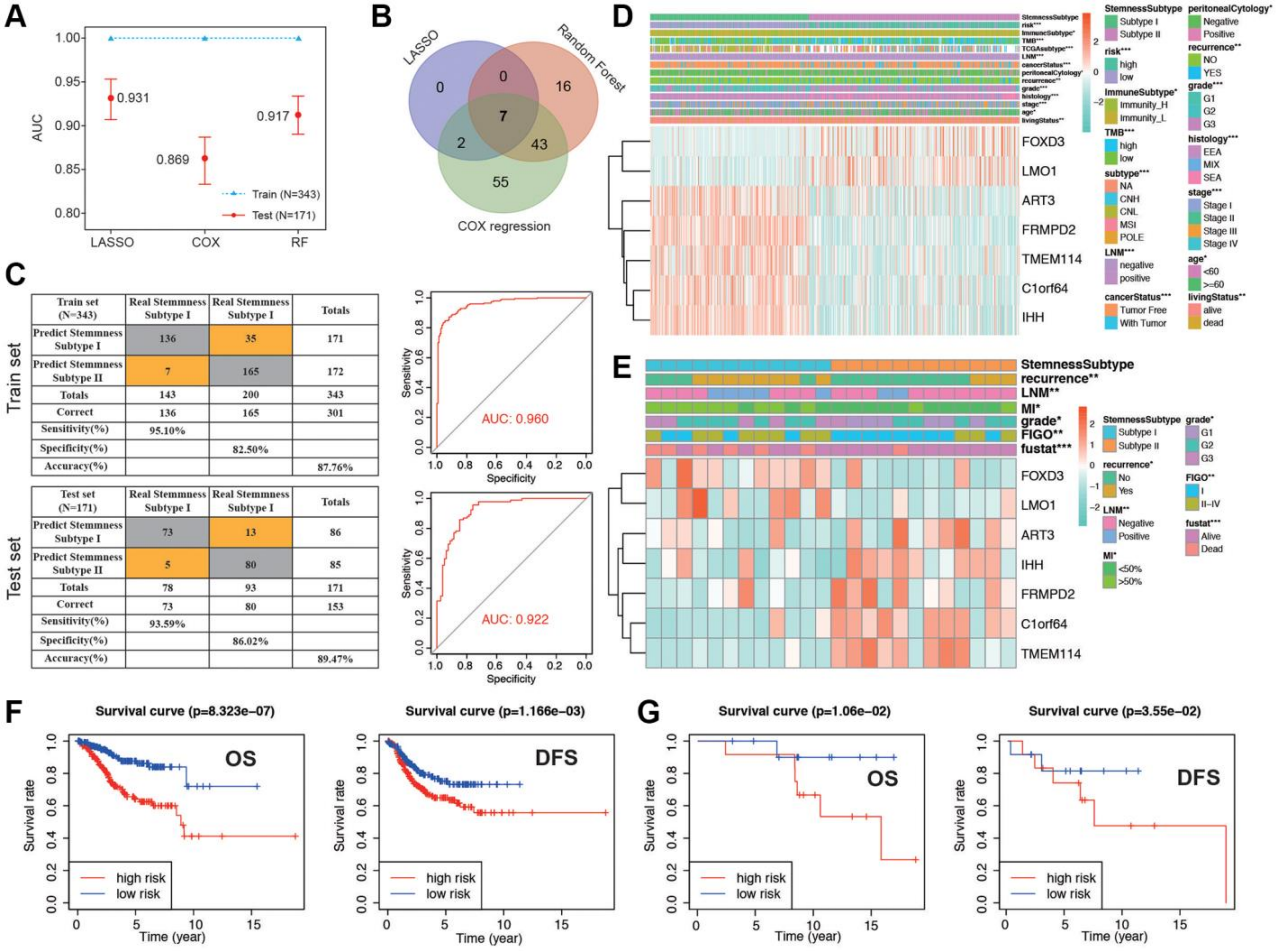
**Establishment and validation of the stemness subtype-based risk signature in TCGA and cohort in our hospital.** (**A**) The performances of three machine-learning algorithms (LASSO, COX and RF) for feature selection were, respectively, evaluated in the training set and validation set. AUCs were generated by ROC analysis. (**B**) Venn diagram showing the common genes of the three machine-learning methods. (**C**) Left panel: confusion matrices of binary results of the Stemness Subtype Predictor for the training set (upper) and validation set (lower). Right panel: ROC curves of the Stemness Subtype Predictor in distinguishing two subtypes in the training set (Upper, AUC = 0.960) and validation set (Lower, AUC = 0.922). (**D**, **E**) The heatmap showing the expression levels of 7 hub genes in the subtype I and subtype II. The distribution of clinicopathological features was compared between the two groups in TCGA cohort and cohorts in our hospital, respectively. (**F**) Kaplan-Meier curve of patients in low- and high-risk groups of OS and DFS in TCGA patients. (**G**) Kaplan-Meier curve of patients in low- and high-risk groups of OS and DFS in patients in our hospital.

We proceeded to split the patients into a training set and a validation set, maintaining a 2:1 ratio. Initially, in the training set, we applied three distinct machine learning algorithms to discern the most crucial stemness subtype-related features based on the expression levels of 1,290 stemness-related DEGs. Simultaneously, time-dependent AUC analysis revealed that the stemness subtype score exhibited substantial predictive value for the Overall Survival (OS) of EC patients within the TCGA dataset. Figure 7A illustrates that LASSO regression yielded the highest AUC, standing at 0.931 in the validation set, as reflected in the corresponding coefficient in Supplementary Table 2. A total of 9, 66, and 107 genes were identified by LASSO, random forest (RF), and Cox regression, respectively (Supplementary Figure 7A–7E). By overlapping the DEGs of the three machine learning methods, we obtained a total of 7 common genes (Figure 7B). Coefficient was shown in Supplementary Table 3. Risk score was calculated with the 7 genes as following: risk score= −(ART3 × 1.120) −(C1orf64 × 0.763) + (FOXD3 × 0.868) −(FRMPD2 × 0.621) −(IHH × 0.263) + (LMO1 × 0.582) −(TMEM114 × 1.434), and divided the patients into low and high groups. Stemness Subtype Predictor also had an excellent performance in discriminating the stemness subtypes as evaluated in the test set (Figure 7C). The expression of the 7 genes subtype I and II patients in the TCGA dataset was also demonstrated in the heatmap (Figure 7D). We observed significant distinctions between subtype I and subtype II groups concerning factors such as risk, immune subtype, Tumor Mutational Burden (TMB), TCGA molecular classification, cancer status, peritoneal cytology, recurrence, grade, histology, stage, age, and survival status. Subsequently, we validated the seven genes along with their associated clinicopathological features within different stemness subtypes in our hospital cohort. As shown in Figure 7E, the patients in our center also confirmed that there was a significant difference in the distribution of clinicopathological characteristics between the two groups. K-M survival analysis indicated that patients in low risk group presented significantly better OS and DFS in both the TCGA and EC cohorts (Figure 7F, 7G). PFS and DSS in TCGA group showed a similar result (Supplementary Figure 7F, 7G). The accuracy of risk model prediction was verified by ROC curve, and the results confirmed that all AUCs of survival prediction was greater than 0.7 (Supplementary Figure 7H–7K). Cohort in our hospital proved that the AUCs of OS and PFS were 0.82 and 0.85, respectively (Supplementary Figure 7L, 7M). Finally, we validated the expression of the 7 genes with normal endometrium and EC tissues by western blot in protein level. The results were shown in Supplementary Figure 8, which is also corresponding with the sequencing results. These findings demonstrate that the classification derived from comprehensive analyses exhibited superior performance, both in the TCGA dataset and among patients in our hospital.

## DISCUSSION

Patients dealing with high-grade, recurrent, and metastatic EC confront considerably unfavorable prognoses. For many years, treatment choices for EC have been far from ideal, presenting significant challenges, until the emergence of immunotherapy provided a glimmer of hope [1, 29]. While reports have highlighted the therapeutic potential of immunotherapy, the search for predictive biomarkers linked to prognosis and the identification of a subgroup with heightened sensitivity to immunotherapy could significantly enhance the outlook for EC patients [3]. Recent years have witnessed substantial advancements in our understanding of the biological attributes of EC, with an increasing emphasis on molecular subtypes rather than histological classifications. In 2013, the TCGA categorized EC into four distinct groups: POLE ultra-mutated, microsatellite instability hypermutated (H-MSI), copy-number low, and copy-number high [30]. The exploration of biological subcategories holds great promise in shaping customized immunotherapy strategies for EC patients in the foreseeable future. TME has been established as a pivotal influencer in modulating gene expression and molecular functions within cancer cells, a factor intricately connected to their receptiveness to immunotherapeutic interventions [31]. Investigating the interplay between EC and the TME, we employed the ESTIMATE algorithm to assess the risk scores associated with immune and stromal cells. Subsequently, we computed the ESTIMATE score, which integrates both cell types. Our next step involved exploring the correlations between these scores and the clinical characteristics of the 514 EC samples. Our findings revealed that lower scores were prevalent in high-grade tumors, implying a potential association between immune molecules in the TME and the degree of EC tumor differentiation. This aligns with the observations made by Jones Nathaniel L et al., suggesting that high-grade tumors exhibit higher immunogenicity compared to low-grade tumors and may consequently be more responsive to immunotherapeutic interventions [32].

In an effort to gain a more comprehensive understanding of the immune and risk scores, we harnessed the power of CIBERSORT algorithms within the R platform to calculate the subtypes of immune cells. Our investigation unveiled notable disparities in the composition of immune cell subtypes when comparing the two risk score groups. Furthermore, GSEA analysis highlighted differences in 14 crucial signaling pathways between the high and low RS groups. Notably, the inhibition of the MAPK signaling pathway was shown to enhance the melanoma immune microenvironment by boosting melanoma antigen expression and suppressing immunosuppressive cytokines [33, 34]. Furthermore, the chemokine signaling pathway plays a significant role in tumor growth. Certain chemokines, including CCR10 and CXCR3, have been demonstrated to play a pivotal role in the proliferation and metastasis of melanoma cells [35].

Introducing a novel stemness index, both the mRNAsi and EREG-mRNAsi indices were computed through the application of an OCLR machine learning algorithm. These indices were found to effectively categorize tumors into specific stemness phenotypes. By analyzing the expression profiles of both EC and normal samples in tandem with their respective stemness indices, we successfully identified five distinct gene modules among the Differentially Expressed Genes (DEGs). Within these modules, we pinpointed hub genes in two that exhibited the most substantial correlations with mRNAsi. Prior research has underlined the robust connections between cancer stemness and critical factors such as cancer metastasis, drug resistance, recurrence, and poor prognosis [36, 37]. Our study conducted comprehensive analyses of cancer stemness in EC patients. These findings support our initial hypothesis that cancer stemness can indeed serve as a valuable biomarker for prognostic predictions in EC. Tumors are known for their significant heterogeneity and intricate compositions. In recent years, there has been a surge in research focused on cancer stem cells. Previous studies have consistently shown that cancer stem cells share key characteristics with stem/progenitor cells, including the ability for self-renewal and multipotent differentiation [38]. While the importance of stemness-related genes is evident, research on therapeutic strategies targeting these genes remains relatively fragmented and lacks comprehensive development. Therefore, there is a compelling need to identify key stemness-related hub genes that could serve as potential therapeutic targets. These stemness-related genes displayed significant connections at both the transcriptional and protein levels, signifying strong biological relationships in their functions. Furthermore, subsequent GO and KEGG analyses unveiled the intricate ties between hub genes associated with stemness and processes involving cell cycle regulation and mitosis. These findings strongly imply their potential involvement in self-renewal and the proliferative properties characteristic of cancer stem cells.

Subsequently, in our quest to understand the interplay between risk scores and immune components, we delved into the potential impact of risk scores on the patterns of immune infiltration and immune scores. Focusing first on immune cells, we acknowledge the diverse array of immune cell types, each carrying distinct roles in the context of anti-tumor responses, immune evasion mechanisms, and the processes of tumor growth, invasion, and metastasis [39–41]. These findings firmly suggest that this signature affects prognosis by interfering with immune cell infiltration in EC. In GO and KEGG analysis, the results revealed that DEGs were enriched in cytoskeletal protein and EMC-related components. The most significant enrichment function and pathway is microtubule-based movement and extracellular matrix structure constituent, respectively, which is predominantly integrin-mediated anchoring junction, located on the basal surface of epithelial cells and serves primarily to integrate the surrounding extracellular matrix (ECM) and actin cytoskeleton [42]. Numerous studies have highlighted the significant role of this pathway in enhancing the migratory, invasive, and adhesive capabilities of cancer cells [43]. By inhibiting the function of key enzymes of this pathway can improve the metastasis and invasion of endometrial cancer cell [44]. Wnt plays a central role as a hub gene in various pathways, including the Wnt signaling pathway and pathways responsible for the regulation of the actin cytoskeleton. These pathways, in turn, play a crucial role in mediating processes such as cell proliferation and differentiation [45].

In the present study, we performed multivariate Cox regression analyses and identified a seven-gene signature including FOXD3, LMO1, ART3, FRMPD2, TMEM114, C1orf64, and IHH. Within these findings, FOXD3 emerged as a pivotal regulator of gene expression distinct to the secretory phase/endometriosis [46, 47]. Notably, FOXD3 exhibited dynamic expression patterns in healthy endometrium and showed significant differential expression in cases of endometriosis [48, 49]. Another study focusing on DNA copy numbers and DNA methylation aberrations in EC have indicated that three potential prognostic markers (KIAA1324, NPR1, and IHH) showed distinct CNV, DNA methylation, and gene expression profiles.

## CONCLUSION

In summary, our study conducted a thorough examination of immune cell profiles within EC tissue samples, leveraging a range of bioinformatics tools. Our analysis uncovered a significant relationship between mRNAsi and diverse clinicopathological attributes, highlighting marked variations in the distribution of mRNAsi across different clinicopathological features and gene mutation profiles. We developed novel prognostic prediction models based on the quantification of infiltrating immune cells and stemness subtypes. These models hold the potential to enhance prognosis assessment and the identification of EC patients who may be particularly well-suited for immunotherapy. Moreover, they exhibited a high degree of accuracy in predicting the prognosis of EC patients. These findings emphasize the considerable promise of immunotherapy in the context of EC and may have important implications for tailoring personalized postoperative follow-up, care, interventions, management, and therapeutic choices for individual EC patients.

## Supplementary Materials

Supplementary Figures

Supplementary Tables

Supplementary File 1

Supplementary File 2

Supplementary File 3

Supplementary File 4

Supplementary File 5
