# Supplementary material for: A stemness-based signature with inspiring indications in discriminating the prognosis, immune response, and somatic mutation of endometrial cancer patients revealed by machine learning
Source: Aging (Albany NY). 2024 Jul 30;16(14):11248–74. doi: 10.18632/aging.205979 (PMC11315399; doi:10.18632/aging.205979)
Supplement: Supplementary Figures [file aging-16-205979-s001.pdf]

SUPPLEMENTARY FIGURES

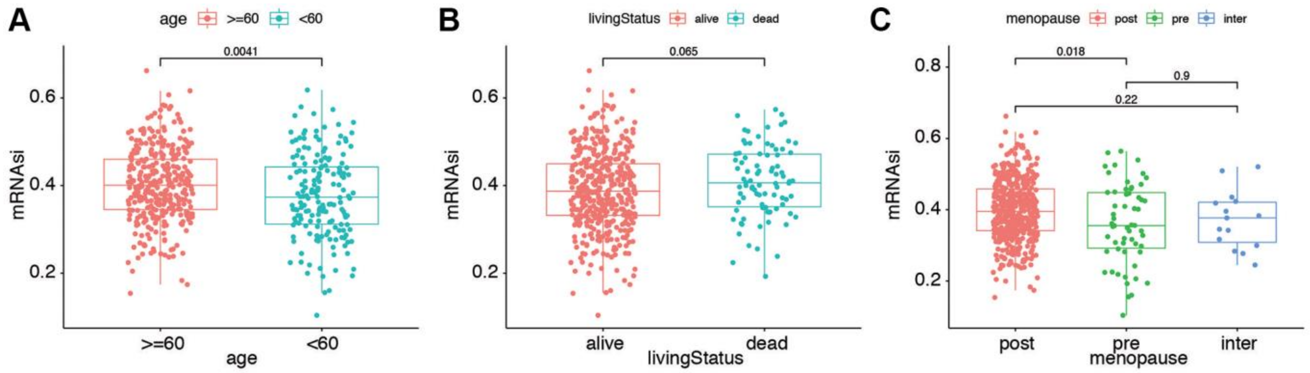

Supplementary Figure 1. (**A**–**C**) Distribution of mRNAsi in different age, living status, and menopausal status.

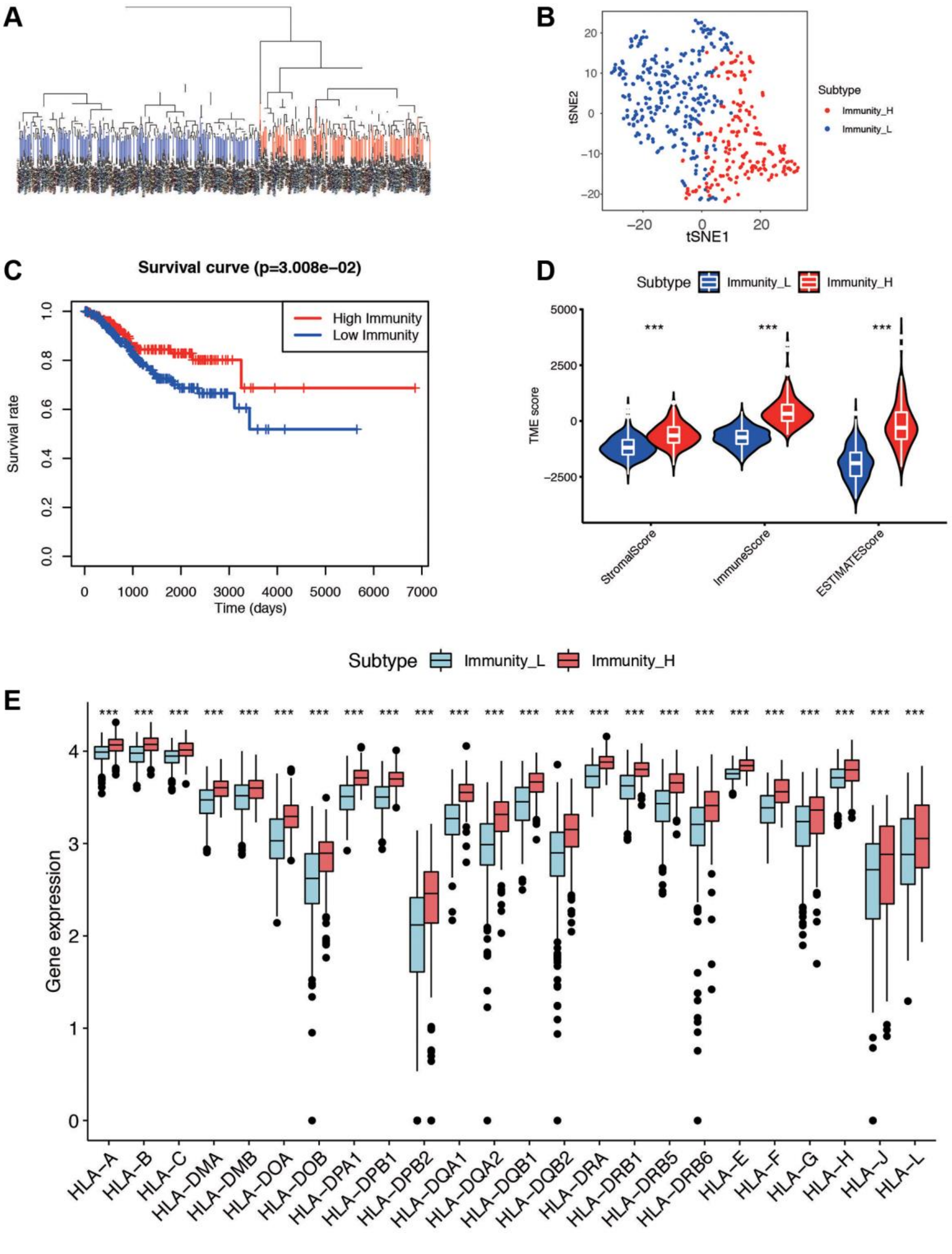

Supplementary Figure 2. (**A**) Unsupervised clustering for 514 EC patients from the TCGA-EC cohort. Patients were classified into two cohort according to the transcriptome data. (**B**) PCA analysis of the two clusters based on the ssGSEA. (**C**) Kaplan-Meier Survival analysis indicates that the two clusters had significantly different survival rates (p = 0.03). (**D**) Difference in Stromal score, Immune score, and ESTIMATE score of samples from two clusters. (**E**) Comparison of the expression levels of HLA genes between two clusters in TCGA.

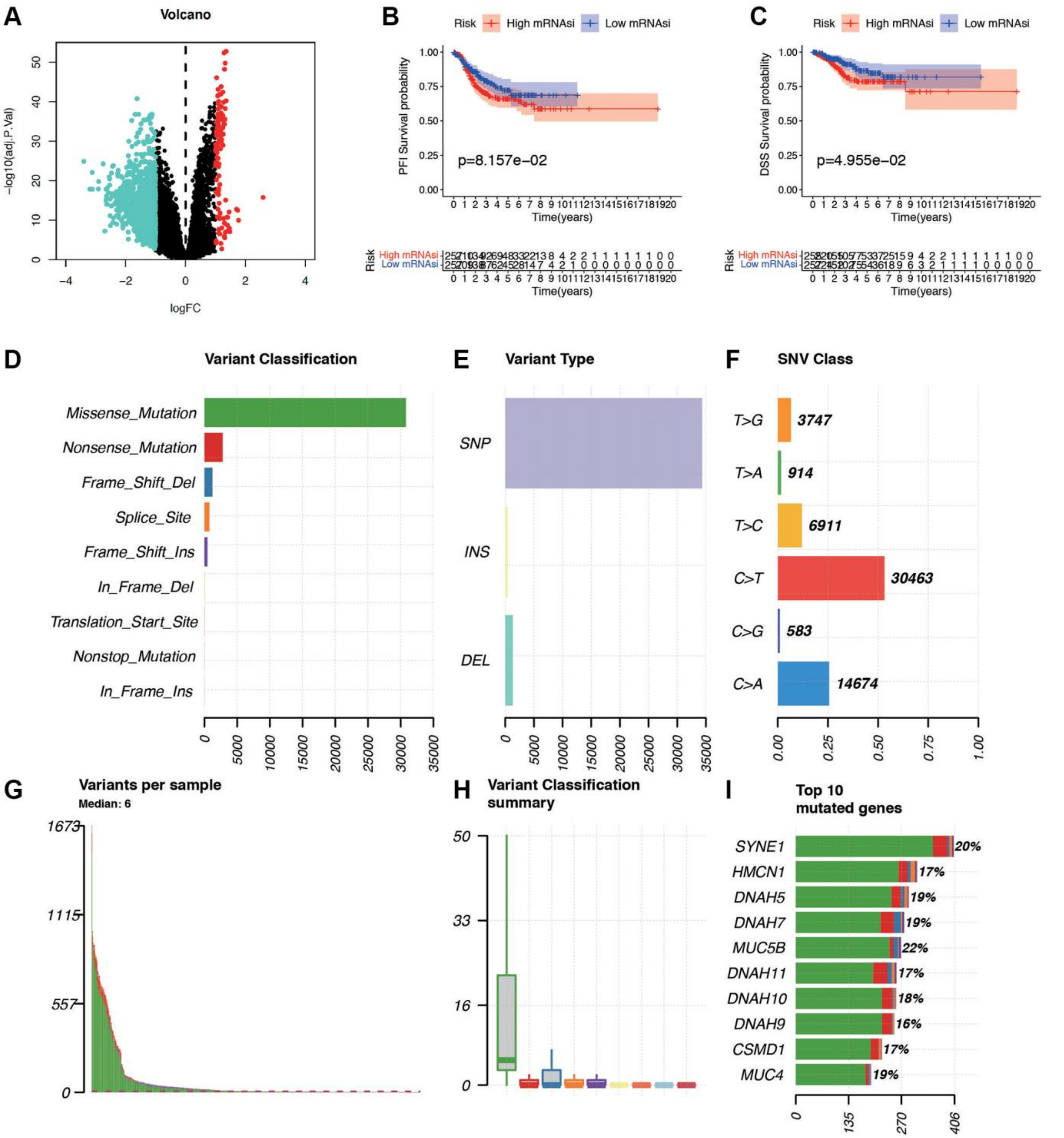

Supplementary Figure 3. (**A**) Volcano plot showing the DEGs between low and high mRNAsi groups. (**B**, **C**) PFS and DSS analysis of patients in different mRNAsi groups. (**D**) Classification and frequency of mutation types. (**E**) Frequency of variant types. (**F**) Frequency of SNV classes. (**G**, **H**) Tumor mutation burden in specific samples. (**I**) The top 10 mutated genes in EC samples.

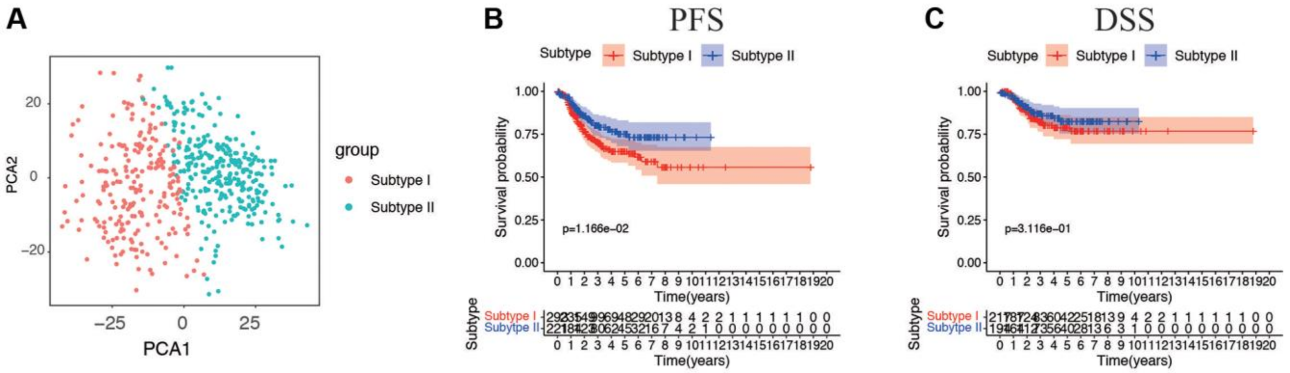

Supplementary Figure 4. (**A**) PCA analysis of patients in Stemness Subtype I and Subtype II. (**B**, **C**) PFS and DSS for patients in different subtypes. The difference is significant in PFS (p = 0.011) but not in DSS (p > 0.05).

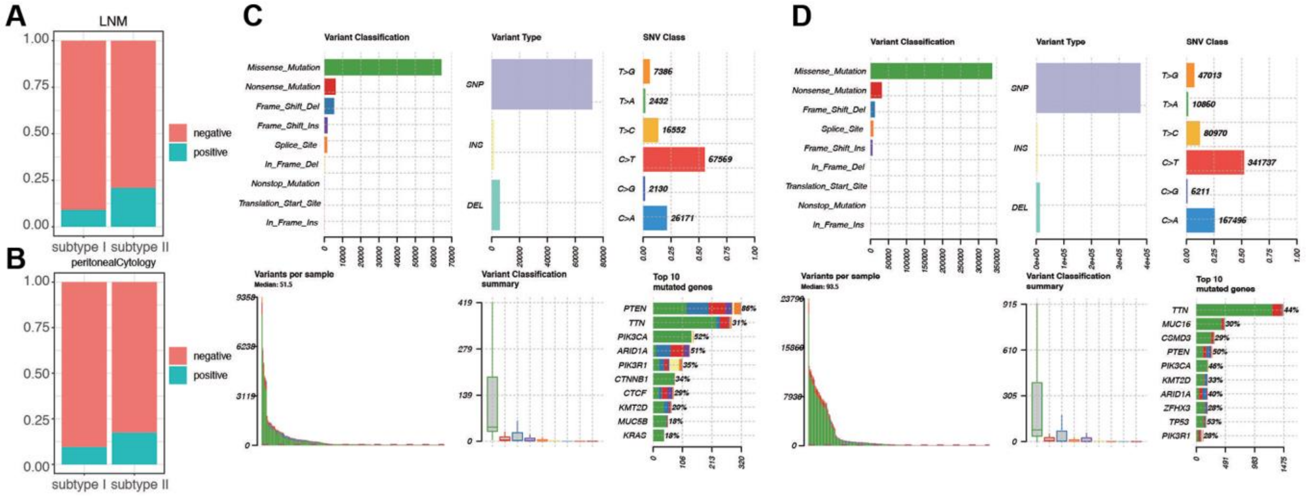

Supplementary Figure 5. (**A**, **B**) Proportions of different lymph node metastasis (LNM) and peritoneal cytology in subtype I and subtype II. (**C**, **D**) Cohort summary plot displaying distribution of variants according to variant classification, type and SNV class in subtype I and subtype II. Bottom part (from left to right) indicates mutation load for each sample, variant classification type. A stacked bar plot shows top ten mutated genes.

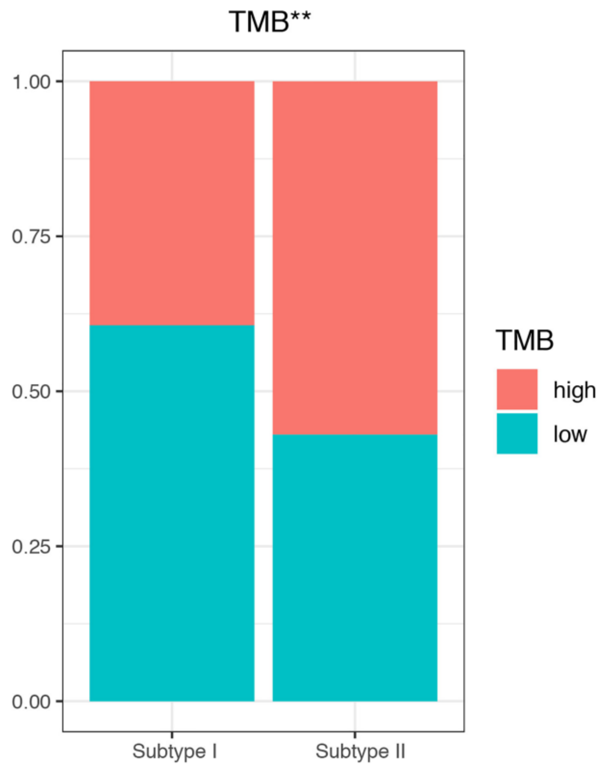

Supplementary Figure 6. Proportions of low and high-TMB in different subtype.

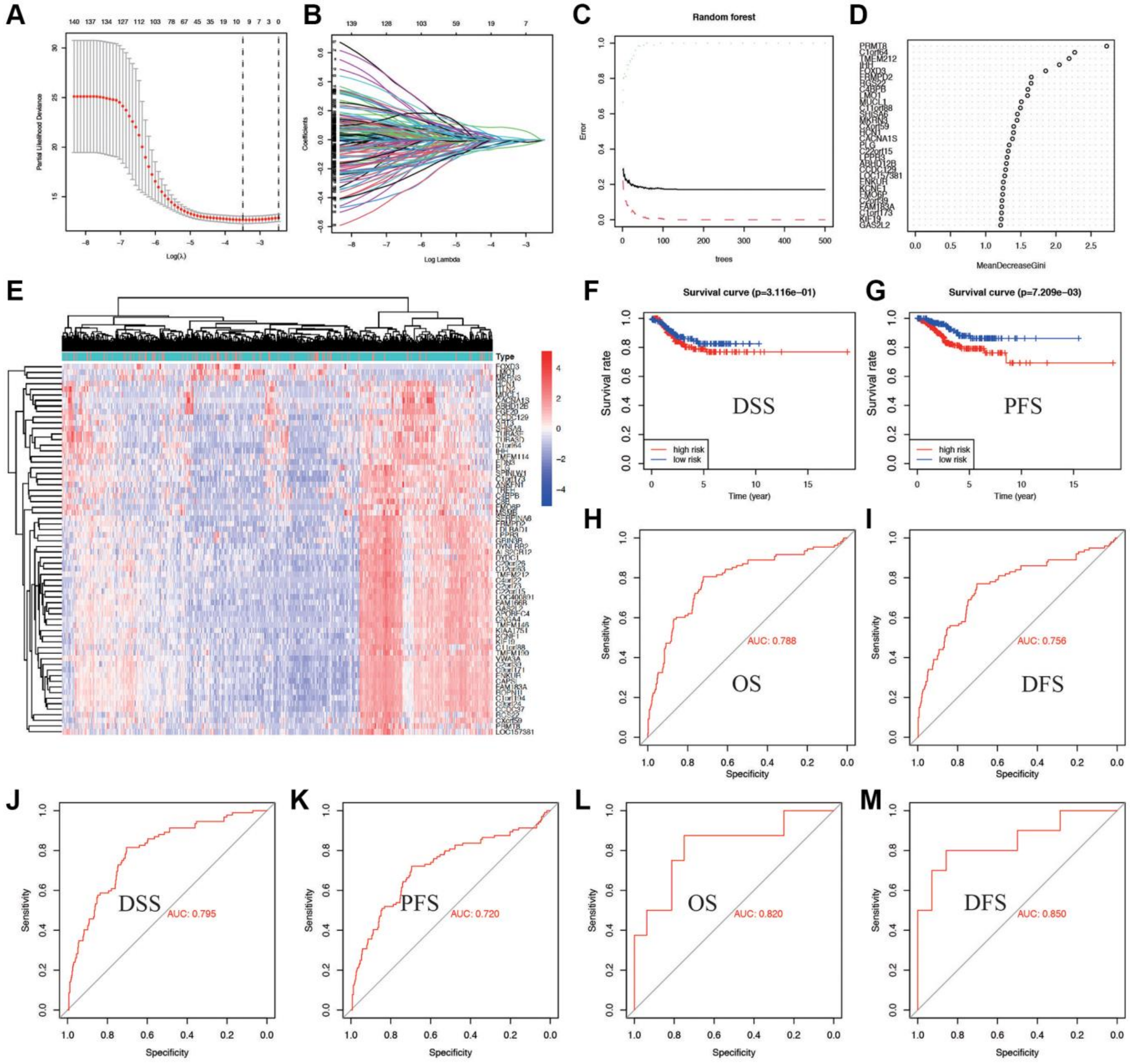

Supplementary Figure 7. (**A**) Distribution of LASSO coefficients for 145 related genes. (**B**) Plots of the cross-validation error rates. The dashes signify the value of the minimal error and greater λ value. (**C**, **D**) Error and mean decrease Gini value of random forest analysis. (**E**) Heatmap of selected genes in different group by RF. (**F**, **G**) Based on the Kaplan-Meier survival analysis, DSS and PFS was significantly higher in the low-risk score group than the high groups. (**H**–**K**) Area under the ROC curve of OS (AUC = 0.788), DFS (AUC = 0.756), PFS (AUC = 0.795), and DSS (AUC = 0.720) in TCGA cohort. (**L**, **M**) Area under the ROC curve of OS (AUC = 0.820), DFS (AUC = 0.850) in cohort in our hospital.

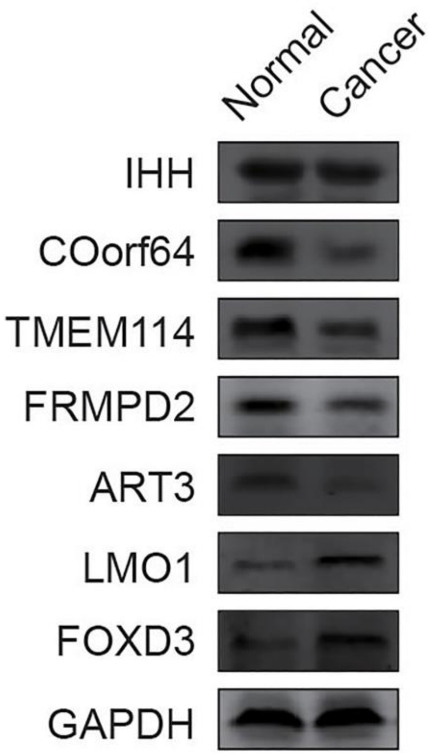

Supplementary Figure 8. Validation of the expression of the 7 genes in normal endometrium and endometrial cancer tissues by western blot.
