# Supplementary material for: A stemness-based signature with inspiring indications in discriminating the prognosis, immune response, and somatic mutation of endometrial cancer patients revealed by machine learning
Source: Aging (Albany NY). 2024 Jul 30;16(14):11248–74. doi: 10.18632/aging.205979 (PMC11315399; doi:10.18632/aging.205979)
Supplement: Supplementary Tables [file aging-16-205979-s002.pdf]

SUPPLEMENTARY TABLES

Supplementary Table 1. Characteristics of patients in TCGA and cohort in our hospital.

Variables	TCGA	Validation
	**Mean + SD**	**Mean + SD**
OS (day)	999.3 ± 854.5	1562 ± 683.6
Age (year)	64.2 ± 11.0	60.7 ± 15.2
Living status	*N* (%)	*N* (%)
Alive	343 (82.4%)	16 (66.7%)
Death	73 (17.5%)	8 (33.3%)
**Diabetes**		
No	286 (68.8%)	17 (70.8%)
Yes	130 (31.2%)	7 (29.2%)
**Hypertension**		
No	159 (38.2%)	12 (50.0%)
Yes	257 (61.8%)	12 (50.0%)
**Menopausal status**		
Premenopausal status	66 (15.9%)	7 (29.2%)
Postmenopausal status	350 (84.1%)	17 (70.8%)
**Tumor grade**		
G1	61 (14.7%)	
G2	89 (21.4%)	19 (79.2%)
G3	266 (63.9%)	5 (20.8%)
**FIGO stage**		
Stage I	255 (61.3%)	13 (54.2%)
Stage II	42 (10.1%)	3 (12.5%)
Stage III	95 (22.8%)	6 (25.0%)
Stage IV	24 (5.8%)	2 (8.4%)
**Histological type**		
EEA	301 (72.4%)	21 (87.5%)
Other types	115 (27.6%)	3 (12.5%)
**Recurrence**		
No	331 (79.6%)	12 (50.0%)
Yes	85 (20.4%)	12 (50.0%)
**Peritoneal cytology**		
Negative	344 (82.7%)	18 (75.0%)
Positive	72 (17.3%)	6 (25.0%)
**LNM**		
Negative	302 (72.6%)	19 (79.17%)
Positive	114 (27.4%)	5 (20.83%)

Abbreviations: TCGA: The Cancer Genome Atlas; OS: overall survival; SD: standard deviation; G: grade; EEA: endometrioid endometrial adenocarcinoma; LNM: lymph node metastasis. In histological types, the other type includes serous endometrial adenocarcinoma and mixed serous and endometrioid.

Supplementary Table 2. Seven genes and corresponding coefficient value in LASSO regression.

Gene names	Coefficient
ART3	–0.0107606
C1orf64	–0.0524939
FOXD3	0.081842
FRMPD2	–0.0075949
FRMPD2L1	–0.0223019
IHH	–0.0060432
LMO1	0.01754824
SERPIND1	–0.0166729
TMEM114	–0.0305824

Supplementary Table 3. Multivariate logistic regression analysis of the seven genes selected by multiple machine learning algorithms.

Variable	B	HR (95%CI)	*p*-value
ART3	–1.120	0.810 (0.719–0.912)	5.28E-04
C1orf64	–0.763	0.845 (0.786–0.908)	5.28E-06
FOXD3	0.868	1.237 (1.129–1.354)	4.33E-06
FRMPD2	–0.621	0.833 (0.761–0.912)	8.02E-05
IHH	–0.263	0.880 (0.834–0.929)	4.40E-06
LMO1	0.528	1.158 (1.073–1.251)	1.65E-06
TMEM114	–1.434	0.753 (0.656–0.865)	5.82E-05
