# Supplementary material for: A stemness-based signature with inspiring indications in discriminating the prognosis, immune response, and somatic mutation of endometrial cancer patients revealed by machine learning
Source: Aging (Albany NY). 2024 Jul 30;16(14):11248–74. doi: 10.18632/aging.205979 (PMC11315399; doi:10.18632/aging.205979)
Supplement: Supplementary File 5 [file aging-16-205979-s007.docx]

**Supplementary File 5. R code used in this study.**

if (!requireNamespace("BiocManager", quietly = TRUE))

install.packages("BiocManager")

BiocManager::install("ComplexHeatmap")

#???ð?

library(ComplexHeatmap)

riskFile="risk.txt" #?????ļ?

cliFile="clinical.txt" #?ٴ??????ļ?

setwd("/Users/jinjiayang/Desktop/BIB/1A/5.cliHeatmap")

#??ȡ?????ļ?

risk=read.table(riskFile, header=T, sep="\t", check.names=F, row.names=1)

risk=risk[order(risk$riskScore),]

#??ȡ?ٴ??????ļ?

cli=read.table(cliFile,sep="\t",header=T,check.names=F,row.names=1)

#?ϲ?????

samSample=intersect(row.names(risk), row.names(cli))

risk=risk[samSample,"risk",drop=F]

cli=cli[samSample,,drop=F]

rt=cbind(risk, cli)

#?ٴ??????Է??????õ??????Ա???

sigVec=c("Risk")

for(clinical in colnames(rt[,2:ncol(rt)])){

data=rt[c("risk", clinical)]

colnames(data)=c("riskScore", "clinical")

data=data[(data[,"clinical"]!="unknow"),]

tableStat=table(data)

stat=chisq.test(tableStat)

pvalue=stat$p.value

Sig=ifelse(pvalue<0.001,"***",ifelse(pvalue<0.01,"**",ifelse(pvalue<0.05,"*","")))

sigVec=c(sigVec, paste0(clinical, Sig))

#print(paste(clinical, pvalue, Sig, sep="\t"))

}

colnames(rt)=sigVec

#??????ͼע??

#rt=rt[apply(rt,1,function(x)any(is.na(match('unknow',x)))),,drop=F]

bioCol=c("#0066FF","#FF9900","#FF0000","#ed1299", "#0dbc21", "#246b93", "#cc8e12", "#d561dd", "#c93f00",

"#ce2523", "#f7aa5d", "#9ed84e", "#39ba30", "#6ad157", "#373bbf", "#a1ce4c", "#ef3bb6", "#d66551",

"#1a918f", "#7149af", "#ff66fc", "#2927c4", "#57e559" ,"#8e3af4" ,"#f9a270" ,"#22547f", "#db5e92",

"#4aef7b", "#e86502", "#99db27", "#e07233", "#8249aa","#cebb10", "#03827f", "#931635", "#ff523f",

"#edd05e", "#6f25e8", "#0dbc21", "#167275", "#280f7a", "#6373ed", "#5b910f" ,"#7b34c1" ,"#0cf29a" ,"#d80fc1",

"#dd27ce", "#07a301", "#ddd53e", "#391c82", "#2baeb5","#925bea", "#09f9f5", "#63ff4f")

colorList=list(Risk=c("low"="green", "high"="red"))

j=0

for(cli in colnames(rt[,2:ncol(rt)])){

cliLength=length(levels(factor(rt[,cli])))

cliCol=bioCol[(j+1):(j+cliLength)]

j=j+cliLength

names(cliCol)=levels(factor(rt[,cli]))

cliCol["unknow"]="grey75"

colorList[[cli]]=cliCol

}

#??????ͼ

ha=HeatmapAnnotation(df=rt, col=colorList)

zero_row_mat=matrix(nrow=0, ncol=nrow(rt))

Hm=Heatmap(zero_row_mat, top_annotation=ha)

#??????ͼ

pdf(file="heatmap.pdf", width=7, height=5)

draw(Hm, merge_legend = TRUE, heatmap_legend_side = "bottom", annotation_legend_side = "bottom")

dev.off()

heatmap

setwd("/Users/jinjiayang/Desktop/BIB/Figure1/1B") #???ù???Ŀ¼

rt=read.table("clusterCliExp.txt",sep="\t",header=T,row.names=1,check.names=F) #??ȡ?ļ?

outpdf="heatmap.pdf"

library(pheatmap)

Type=read.table("clusterCliGroup.Sig.txt",sep="\t",header=T,row.names=1,check.names=F)

Type=Type[order(Type$risk),]

rt=rt[,row.names(Type)]

pdf(outpdf,height=2.2,width=10)

pheatmap(rt, annotation=Type,

color = colorRampPalette(c("white", "grey", "springgreen4"))(50),

cluster_cols =F,

fontsize=8,

fontsize_row=9,

show_colnames=F,

fontsize_col=3)

dev.off()
